# Awareness of Palestinian Women About Breast Cancer Risk Factors: A National Cross-Sectional Study

**DOI:** 10.1200/GO.22.00087

**Published:** 2022-12-12

**Authors:** Mohamedraed Elshami, Faten Darwish Usrof, Mohammed Alser, Ibrahim Al-Slaibi, Heba Mahmoud Okshiya, Roba Jamal Ghithan, Nouran Ramzi Shaban Shurrab, Islam Osama Ismail, Ibtisam Ismail Mahfouz, Aseel AbdulQader Fannon, Malak Ayman Mousa Qawasmi, Mona Radi Mohammad Hawa, Narmeen Giacaman, Manar Ahmaro, Rula Khader Zaatreh, Wafa Aqel AbuKhalil, Noor Khairi Melhim, Ruba Jamal Madbouh, Hala Jamal Abu Hziema, Raghad Abed-Allateef Lahlooh, Sara Nawaf Ubaiat, Nour Ali Jaffal, Reem Khaled Alawna, Salsabeel Naeem Abed, Bessan Nimer Ali Abuzahra, Aya Jawad Abu Kwaik, Mays Hafez Dodin, Raghad Othman Taha, Dina Mohammed Alashqar, Roaa Abd-al-Fattah Mobarak, Tasneem Smerat, Nasser Abu-El-Noor, Bettina Bottcher

**Affiliations:** ^1^ Division of Surgical Oncology, Department of Surgery, University Hospitals Cleveland Medical Center, Cleveland, OH; ^2^ Ministry of Health, Gaza, Palestine; ^3^ Department of Medical Laboratory Sciences, Faculty of Health Sciences, Islamic University of Gaza, Gaza City, Palestine; ^4^ Almakassed Hospital, Jerusalem, Palestine; ^5^ Al-Shifa Hospital, Gaza, Palestine; ^6^ Faculty of Medicine, Al-Quds University, Palestine; ^7^ Faculty of Medicine, Al Azhar University-Gaza, Gaza, Palestine; ^8^ Faculty of Medicine, Islamic University of Gaza, Gaza, Palestine; ^9^ Department of Medical Laboratory Sciences, Hebron University, Hebron, Palestine; ^10^ Tulkarem Governmental Hospital, Tulkarem, Palestine; ^11^ Caritas Baby Hospital, Bethlehem, Palestine; ^12^ Department of Pharmacy, An-Najah National University, Nablus, Palestine; ^13^ Faculty of Dentistry, Al-Quds University, Jerusalem, Palestine; ^14^ Faculty of Medicine and Health Sciences, Palestine Polytechnic University, Hebron, Palestine; ^15^ Faculty of Nursing, Islamic University of Gaza, Gaza, Palestine

## Abstract

This study aimed to assess awareness of Palestinian women about breast cancer (BC) age-related and lifetime risks and its risk factors and to identify factors associated with good awareness.

**MATERIALS AND METHODS:**

Adult women were recruited from government hospitals, primary health care centers, and public spaces in 11 governorates in Palestine. Recognition of 14 BC risk factors was assessed using a translated-into-Arabic version of the validated BC awareness measure. The level of BC risk factor awareness was determined on the basis of the number of risk factors recognized: poor (0-4), fair (5-9), and good (10-14).

**RESULTS:**

Of 6,269 potential participants approached, 5,434 agreed and completed the questionnaire (response rate = 86.7%). A total of 5,257 questionnaires were included: 2,706 from the West Bank and Jerusalem and 2,551 from the Gaza Strip. Only 173 participants (3.3%) recognized the age-related risk of BC. More than one quarter (n = 1,465; 27.9%) recognized the lifetime risk of BC. The most recognized modifiable risk factor was not breastfeeding (n = 4,937; 93.9%), whereas the least recognized was having children later on in life or not at all (n = 1,755; 33.4%). The most recognized nonmodifiable risk factor was radiation exposure (n = 4,579; 87.1%), whereas the least recognized was starting the periods at an early age (n = 1,030; 19.6%). In total, 2,024 participants (38.4%) demonstrated good BC risk factor awareness. Participants from the Gaza Strip had a higher likelihood than participants from the West Bank and Jerusalem to have good awareness (42.0% *v* 35.2%). Age ≥ 40 years, postsecondary education, and visiting hospitals and primary health care centers were all associated with an increase in the likelihood of having good BC risk factor awareness.

**CONCLUSION:**

The awareness of BC risk factors was suboptimal. These findings highlight the need for implementing health education programs combined with consistent use of ad hoc opportunities to raise awareness by health care providers.

## INTRODUCTION

Breast cancer (BC) is the most common cancer among women and the second most common cancer globally, with approximately 2.3 million cases diagnosed annually.^1^ The incidence of BC has substantially increased in recent years among Arab women in the Eastern Mediterranean Region, and the incidence is expected to double by 2030.^2^ In Palestine, BC is the most prevalent cancer among women, accounting for 32.0% of cancer diagnoses in the West Bank and Jerusalem (WBJ) and 18% of these in the Gaza Strip.^3^ In 2018, BC was the third leading cause of cancer-related mortality in Palestine with 12.0%, after lung (20.0%) and colon cancer (13.0%).^4^

**CONTEXT**

**Key Objective**
Breast cancer (BC) is a significant cause of cancer-related morbidity and mortality in Palestine, making it a serious public health concern. Therefore, this national study assessed the awareness of Palestinian women about BC risk factors and examined the sociodemographic factors associated with good awareness.
**Knowledge Generated**
The awareness of BC risk factors was relatively low, with only 38.4% of participants displaying good awareness. Living in the Gaza Strip, age ≥ 40 years, postsecondary education, and visiting hospitals and primary health care centers were all associated with good awareness.
**Relevance**
Poor public awareness of BC risk factors may contribute to longer delays in seeking medical advice, leading to diagnosis at advanced stages and eventually lower survival rates. Educational interventions aiming to improve BC awareness are needed and should be tailored to address the knowledge gaps among Palestinian women.

BC is highly treatable when detected early.^5^ Unfortunately, more than 60.0% of BC cases in Palestine are diagnosed at later stages, reducing the chance of survival.^3^ One of the contributing factors to this delayed diagnosis could be poor knowledge of the warning signs of BC and limited awareness of risk factors.^6^ Lifetime risk assesses the likelihood of developing BC at any point after exposure to risk factors, regardless of when the incident occurred.^7^

Modifiable risk factors of BC include no history of breastfeeding^8^; obesity^9^; physical inactivity^10^; use of some medications, such as oral contraceptives^11^; smoking^12^; and having children later on in life or not at all.^13^ By contrast, nonmodifiable risk factors include female sex, older age, early menarche, late menopause, family or personal history of BC, and radiation exposure.^14-17^

A previous study from the Gaza Strip showed low awareness of symptoms and risk factors of BC.^18^ Continuous education programs can raise BC awareness, which could facilitate early diagnosis.^19^ However, there is a need to evaluate the baseline knowledge among women in the Palestinian community. Therefore, this study aimed to (1) assess awareness of Palestinian women about age-related and lifetime risks of BC and its risk factors, (2) compare the awareness among women from the Gaza Strip versus the WBJ, and (3) examine the factors associated with good BC risk factor awareness.

## MATERIALS AND METHODS

### Study Design and Population

Detailed methods are as published.^19^ Briefly, a national cross-sectional study was conducted between July 2019 and March 2020 in the two main geographical areas of Palestine: the Gaza Strip and the WBJ. Adult Palestinian women (age ≥ 18 years) were the target population. Women visiting Palestinian government hospitals; primary health care centers; and public venues, such as malls, restaurants, markets, gardens, transportation hubs, churches, and mosques, were recruited. Exclusion criteria were working or studying in a health-related field, non-Palestinian citizenship, and attending oncology departments or clinics.

### Sampling Methods

Eligible women were recruited using a convenience sampling method from the specified data collecting sites at 11 governorates in Palestine. This facilitated recruitment of women from a wide variety of backgrounds to the study cohort and was intended to make it resemble the Palestinian community.^19-24^

### Questionnaire and Data Collection

A modified version of the BC Awareness Measure (BCAM) was used for data collection. The BCAM is a validated questionnaire that was created to assess public awareness of BC.^25^ A back-to-back translation was performed, where the original BCAM was translated into Arabic by two bilingual experts and the Arabic version was subsequently translated back into English by two additional bilingual experts. The Arabic version of the BCAM was then evaluated by five professionals in the areas of BC, public health, and survey design for content validity and translation accuracy. This was followed by running a pilot study (n = 35) to assess the clarity of the Arabic questionnaire. Cronbach's Alpha was used to examine the internal consistency of the Arabic questionnaire, which reached an acceptable value of 0.72.

The questionnaire comprised three sections. The first section described the sociodemographic factors of study participants. The second section evaluated the awareness of age-related and lifetime risks to develop BC. The third section evaluated the recognition of 14 BC risk factors on the basis of a 5-point Likert scale (1 = strongly disagree and 5 = strongly agree). Of those 14 factors, eight were adapted from the original BCAM^25^ and six (not breastfeeding, long-term use of the contraceptive pill, smoking, passive smoking, consumption of fatty foods, and radiation exposure) were added as they were deemed to be important to assess the awareness of these additional factors in the Palestinian community.^22,23,26-30^

The electronic tool Kobo Toolbox, which can be used by smartphones, was used to collect data.^31^ In a face-to-face interview with the data collector present, recruited women were asked to complete the questionnaire. A special training was conducted to help female data collectors, who had a medical background, learn using Kobo Toolbox, approach study participants, and facilitate the completion of the questionnaire.

### Statistical Analysis

In Palestine, women are first invited to get a BC screening at the age of 40 years.^32^ On the basis of this cutoff, age was divided into two categories: 18-39 years and ≥ 40 years. Menarche, the age at which periods begin, was also divided into three categories: early (under 11 years), normal (between 11 and 15 years), and late (beyond 16 years).^33^ Since 1,450 New Israeli Shekel (NIS) (about $450 US dollars) was the minimum wage of Palestinian employees,^34^ it was chosen to categorize monthly income into two categories: < 1,450 and ≥ 1450 NIS.

The median (interquartile range) was used to describe continuous, non-normally distributed variables, and the Kruskal-Wallis test was used for baseline comparisons. Frequencies and percentages were used to describe categorical variables, and Pearson's chi-square test was used for baseline comparisons.

On the basis of the original BCAM,^25^ a 70-year-old woman and one in eight women were considered as correct answers for age-related and lifetime risks of BC, respectively. Recognition of both the age-related and lifetime risks was described using frequencies and percentages. Bi- and multivariable logistic regression analyses were used to test the association between participant characteristics and recognition of the age-related and lifetime risks of BC.

Responses with strongly agree and agree were considered correct for questions on the basis of a 5-point Likert scale, whereas those with strongly disagree, disagree, and not sure were considered incorrect. BC risk factors were categorized into two main categories: modifiable and nonmodifiable risk factors. Frequencies and percentages were used to describe the recognition of each BC risk factor with comparisons performed using Pearson's chi-square test. This was followed by running bi- and multivariable logistic regression analyses to examine the association between recognizing each BC risk factor and participant characteristics.

To assess the awareness of BC risk factors, a scoring system was used. A similar scoring system was also used in previous studies.^19-24,35^ One point was given for each correctly recognized BC risk factor. The total score (ranging from 0 to 14) was then calculated and categorized into three categories on the basis of the number of BC risk factors recognized: poor (0-4), fair (5-9), and good (10-14). Comparisons in BC risk factor awareness level between the Gaza Strip versus the WBJ were performed using Pearson's chi-square test. This was followed by running bi- and multivariable logistic regression analyses to test the association between having a good awareness level of BC risk factors and participant characteristics.

All multivariable logistic regression analyses were adjusted for age group, menarche, education, occupation, monthly income, place of residence, marital status, having a chronic disease, knowing someone with cancer, and site of data collection. This model was determined a priori on the basis of previous studies.^18,19,36-41^ Results of the bivariable logistic regression analyses are provided in Appendix Tables A1-A4.

Missing data occurred completely at random and were handled using a complete case analysis approach. Data were analyzed using Stata software version 16.0 (StataCorp, College Station, TX).

### Ethical Considerations

This study was approved by the ministry of health's Helsinki Committee in the Gaza Strip, which reviews and approves human studies. In addition, this study was approved by the Palestinian MOH's Human Resources Development department and the Islamic University of Gaza University's Ethics Committee. Before beginning the interview, study participants provided written informed consent. All participants received a thorough explanation of the study before starting the interview, with the emphasis on the fact that participation was entirely voluntary and would not have any impact on the medical care that they would receive. Data confidentiality was maintained throughout the study.

## RESULTS

### Participant Characteristics

Of 6,269 potential participants approached, 5,434 agreed and completed the questionnaire (response rate = 86.7%). A total of 5,257 questionnaires were included in the final analysis (164 had missing data, and 13 did not meet inclusion criteria). Of those 5,257 participants, 2,706 were from the WBJ and 2,551 were from the Gaza Strip. Among all participants, the median age (interquartile range) for was 31.0 years (24.0-43.0 years; Table 1). Participants from the Gaza Strip were younger and had lower monthly income, but suffered from fewer comorbidities than participants from the WBJ. These sociodemographic data are similar to those presented in Table 1 in another study by our group.^19^

**TABLE 1:**
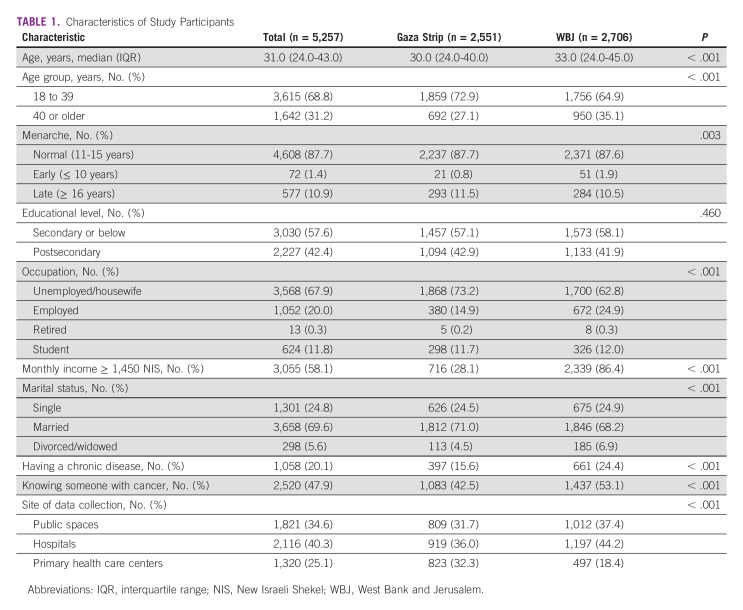
Characteristics of Study Participants

### Recognition of Age-Related and Lifetime Risks of BC

Only 173 participants (3.3%) recognized the age-related risk of BC. Employees, students, and participants recruited from hospitals were more likely to recognize the age-related risk of BC (Table 2). Conversely, participants who knew someone with cancer were less likely to recognize the age-related risk of BC.

**TABLE 2:**
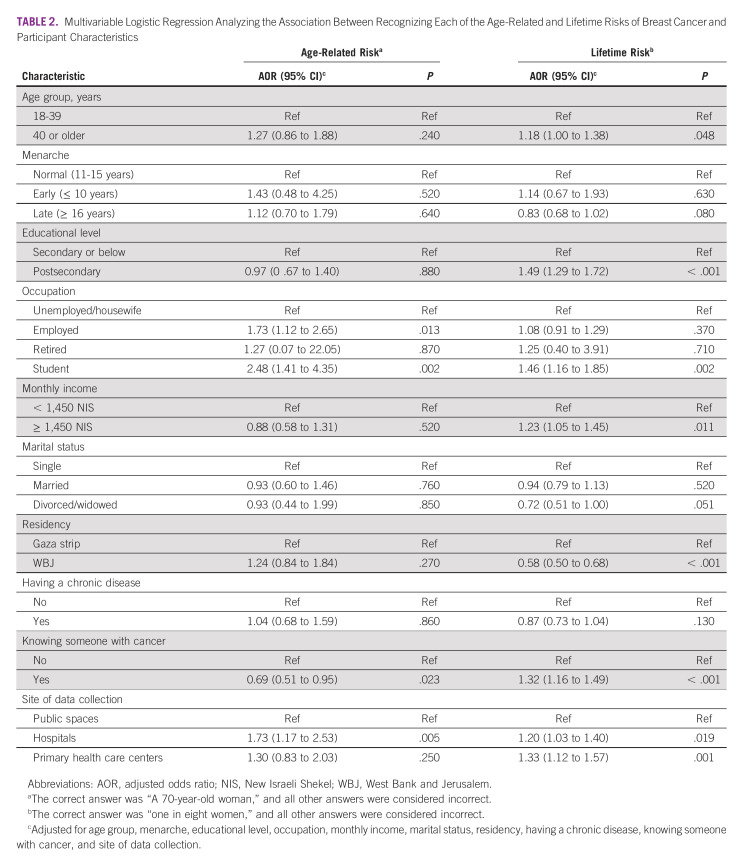
Multivariable Logistic Regression Analyzing the Association Between Recognizing Each of the Age-Related and Lifetime Risks of Breast Cancer and Participant Characteristics

More than one quarter (n = 1,465; 27.9%) recognized the lifetime risk of BC. Participants from the WBJ were less likely than participants from the Gaza Strip to recognize the lifetime risk of BC. By contrast, participants who completed postsecondary education, had a higher monthly income, knew someone with cancer, and visited hospitals or primary health care centers were more likely to recognize the lifetime risk of BC.

### Recognition of BC Risk Factors

Among all participants, the most recognized modifiable BC risk factor was not breastfeeding (n = 4,937; 93.9%) followed by not doing 30 minutes of moderate physical activity 5 times a week (n = 4,851; 92.3%; Table 3). The least recognized modifiable BC risk factors were having children later on in life or not at all (n = 1,755; 33.4%) and being overweight (n = 2,420; 46.0%). The most recognized nonmodifiable BC risk factor was radiation exposure (n = 4,579; 87.1%) followed by having a history of BC (n = 4,227; 80.4%). The least recognized nonmodifiable BC risk factors were starting the periods at an early age (n = 1,030; 19.6%) and having a late menopause (n = 1,346; 25.6%). Trends to recognize both modifiable and nonmodifiable BC risk factors were similar in the Gaza Strip and the WBJ.

**TABLE 3:**
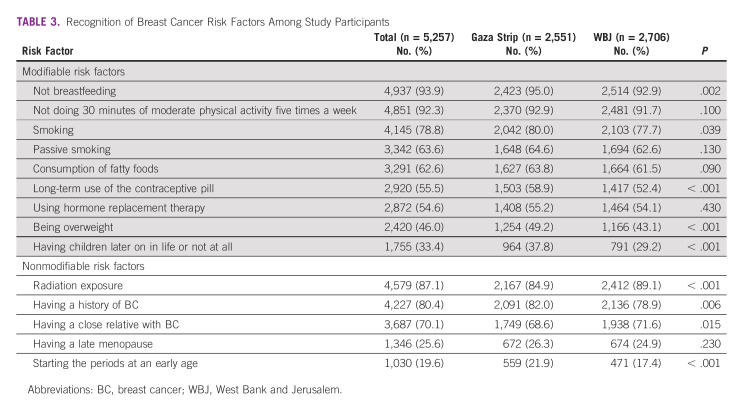
Recognition of Breast Cancer Risk Factors Among Study Participants

### Good BC Risk Factor Awareness and Its Associated Factors

In total, 2024 participants (38.4%) demonstrated good BC risk factor awareness (Table 4). Participants from the Gaza Strip had a higher likelihood than participants from the WBJ to have good awareness (42.0% *v* 35.2%). In the multivariable analysis, age ≥ 40 years, postsecondary education, and visiting governmental hospitals and primary health care centers were all associated with an increase in the likelihood of having good BC risk factor awareness (Table 5). On the other hand, having late menarche and living in the WBJ were associated with a decrease in the likelihood of having good awareness.

**TABLE 4:**
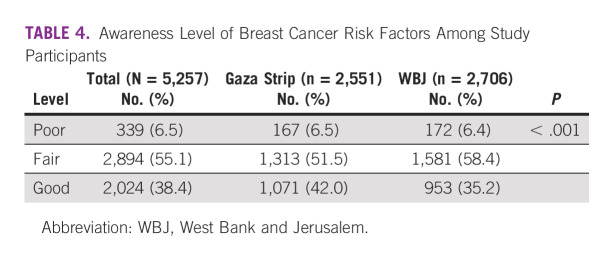
Awareness Level of Breast Cancer Risk Factors Among Study Participants

**TABLE 5:**
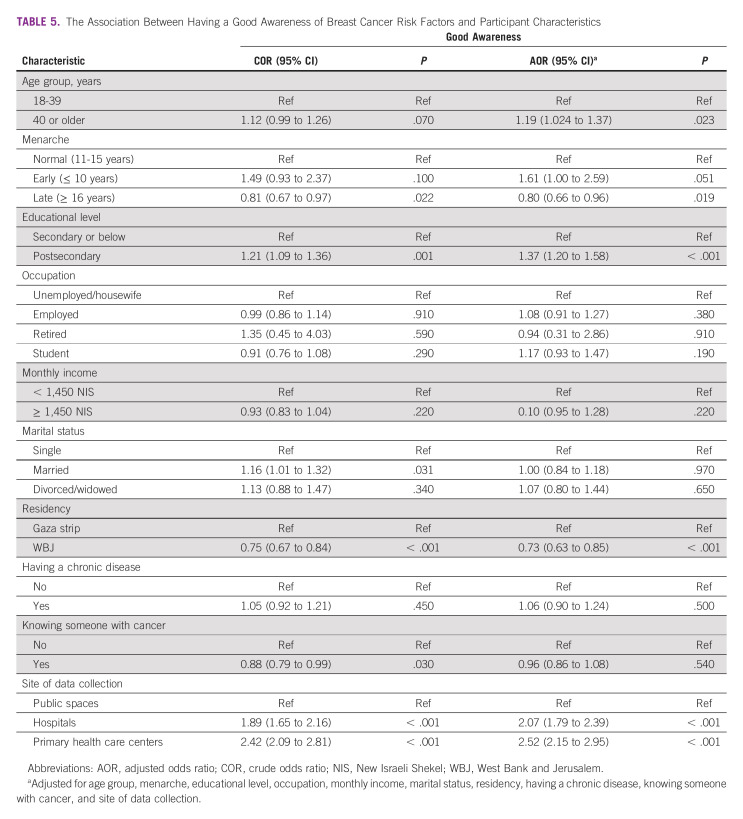
The Association Between Having a Good Awareness of Breast Cancer Risk Factors and Participant Characteristics

### Association Between Recognizing Modifiable BC Risk Factors and Participant Characteristics

In the multivariable analysis, participants recruited from hospitals and primary health care centers were more likely to recognize eight and seven risk factors, respectively, of nine modifiable BC risk factors (Table 6). In addition, participants who completed postsecondary education were more likely to recognize four of the nine modifiable BC risk factors.

**TABLE 6:**
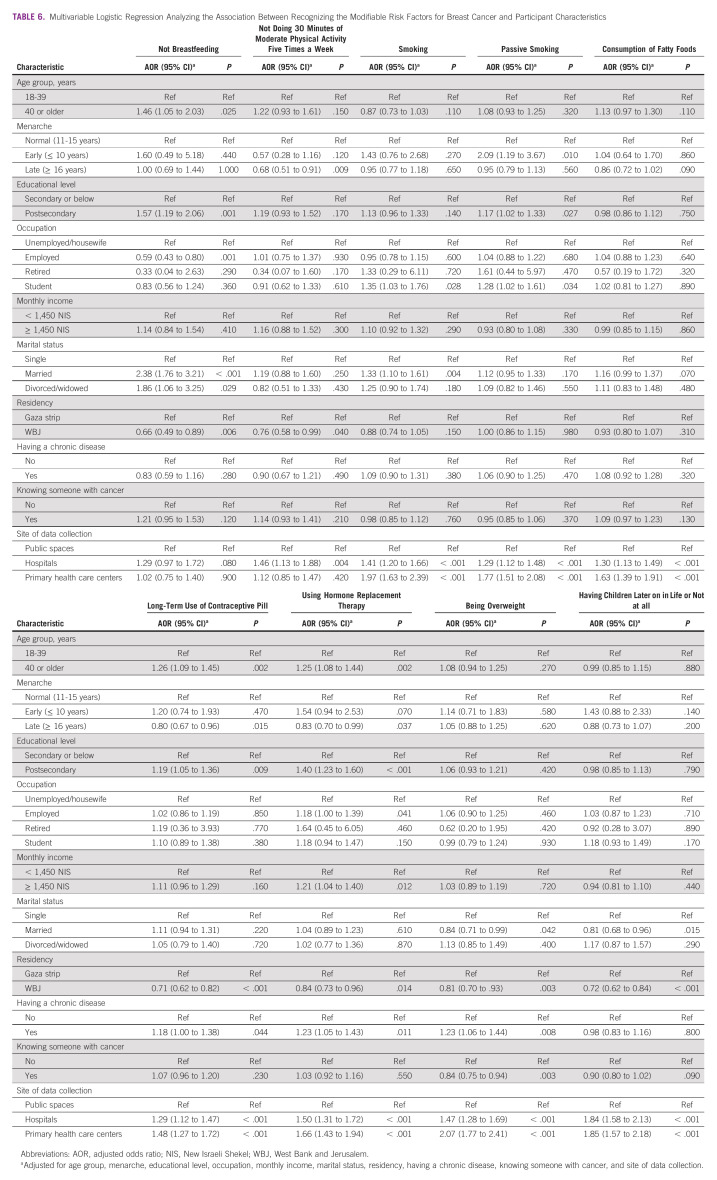
Multivariable Logistic Regression Analyzing the Association Between Recognizing the Modifiable Risk Factors for Breast Cancer and Participant Characteristics

On the contrary, participants from the WBJ were less likely to recognize six of the nine modifiable BC risk factors. Married women were less likely to recognize being overweight (odds ratio [OR], 0.84; 95% CI, 0.71 to 0.99) and having children later on in life or not at all (OR, 0.81; 95% CI, 0.68 to 0.96).

### Association Between Recognizing Nonmodifiable BC Risk Factors and Participant Characteristics

Participants recruited from hospitals had a higher likelihood to recognize all nonmodifiable BC risk factors (Table 7). Furthermore, participants recruited from primary health care centers were more likely to recognize four of the five nonmodifiable BC risk factors. Participants who completed postsecondary education or had a higher monthly income were more likely to recognize three of the five nonmodifiable BC risk factors. By contrast, participants from the WBJ were less likely than participants from the Gaza Strip to recognize having a history of BC (OR, 0.63; 95% CI, 0.52 to 0.75) and starting the periods at an early age (OR, 0.75; 95% CI, 0.63 to 0.89).

**TABLE 7:**
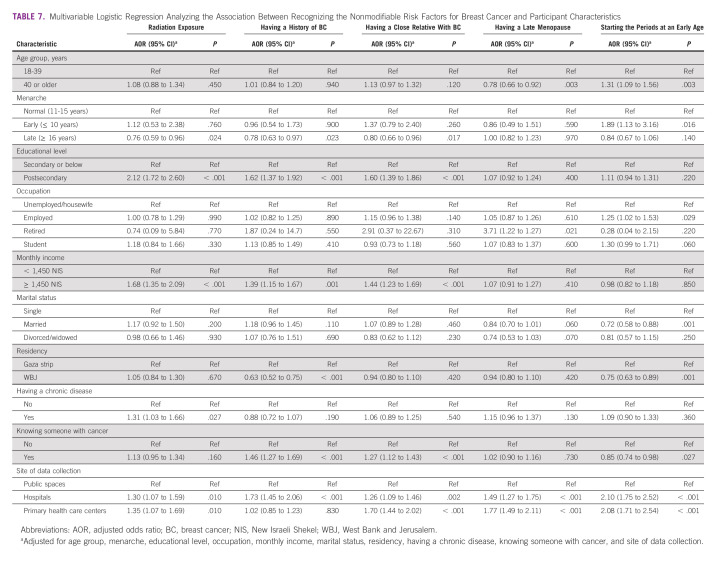
Multivariable Logistic Regression Analyzing the Association Between Recognizing the Nonmodifiable Risk Factors for Breast Cancer and Participant Characteristics

## DISCUSSION

The recognition of age-related and lifetime risks of developing BC was poor in this study, with 3.3% and 27.9% of the participants recognizing them correctly, respectively. Awareness of BC risk factors was also low, with only 38.4% of the participants having good awareness. Compared with participants from the WBJ, participants from the Gaza Strip were more likely to demonstrate good BC risk factor awareness. Age 40 years or older, postsecondary education, and visiting hospitals and primary health care centers were all associated with good BC risk factor awareness. Not breastfeeding and not doing physical activity were the most recognized modifiable risk factors, whereas having children later on in life was the least recognized.

In line with a previous study from the Gaza Strip,^18^ Palestinian women displayed poor awareness of greater risk of BC with increased age in this study. However, lifetime risk for women to get BC was recognized by a larger proportion in both studies with 42.6% in the previous study and only 27.6% in this study.^18^ Poor awareness of age-related and lifetime risks of BC, as in this study, appears to be a global finding in low-income and high-income settings although awareness was shown to be consistently lower in the former.^36,42-45^ Regular awareness campaigns and organized BC screening systems in high-income countries could be contributing factors to this difference. Interestingly, older women, who indicated a higher level of risk awareness, rarely perceived age as a risk factor. Furthermore, older age groups showed lower cancer awareness.^46^ Future educational interventions should be targeting this knowledge gap among older women.

In line with a previous study,^47^ not breastfeeding was the most recognized modifiable BC risk factor in this study. It appears that women are more aware of breastfeeding as a protective factor for BC than other factors. Globally, many initiatives have promoted breastfeeding and included education on the benefits of breastfeeding, which could have contributed to the knowledge acquisition by women on this issue.^48^ This demonstrates the potential impact on awareness by education campaigns and contact with health care professionals, which could be a powerful tool for improving health literacy.^49^

Around 92.0% of the study participants believed that physical inactivity was a risk factor for the development of BC, which was a substantially higher percentage than the 75.0% reported by Lahart et al^50^ from the United Kingdom. However, despite this knowledge, 57.8% of Palestinians are overweight and this might be an average percentage in the international context.^51^ This demonstrates that awareness and knowledge of risk factors alone do not necessarily translate into adoption of healthier lifestyles. Therefore, increased awareness might need to be coupled with effective public health strategies to promote the adoption of healthy lifestyles for greatest impact on reduction of disease burden.

Good awareness of the risk factors of BC is the essence of primary prevention as it might empower women to make healthy lifestyle choices.^18^ Although nonmodifiable risk factors such as female sex, advanced age, and genetic factors retain the greatest impact on BC prevalence and mortality, the control of modifiable risk factors, such as obesity, smoking, and lack of regular exercise, can also reduce prevalence and, thus, BC mortality. It has been estimated that with the control of all modifiable risk factors, a reduction of at most 30% in BC risk could be achieved.^52-54^ In the absence of organized screening programs, delayed BC diagnosis, and lack of access to effective treatment options, as in Palestine, risk factor reduction might be one way of reducing BC mortality.^55-57^ Therefore, good BC risk factor awareness may still play a significant role in reducing BC prevalence and mortality, particularly in low- and middle-income countries like Palestine. This study showed low awareness with only 38.4% of participants displaying good BC risk factor awareness, which is consistent with data from other low- and middle-income countries (such as India^47^ and Nigeria^58^) and high-income countries (such as Iran^59^ and Saudi Arabia^60^). The greatest risk factor, advanced age, was only recognized by 3.3% in this study, compared with 14.0% in the United Kingdom and up to 38.0% in Sweden.^61^ In concordance with a previous study,^43^ older women and those with higher education were more likely to have good BC risk factor awareness. Furthermore, the BC risk factor awareness was significantly greater among women visiting hospitals and primary health care centers, highlighting the important role of health care practitioners in forming patients' health literacy.

Women living in the Gaza Strip were more likely than those living in the WBJ to have good BC risk factor awareness (42.0% *v* 35.2%). This difference might be compounded by the fact that the WBJ has checkpoints and restrictions on internal mobility, even between cities, making it difficult to reach health care facilities.^57^ Another explanation could be the number of women living in rural regions, where the WBJ has a higher proportion, who might have limited access to health care facilities.^62^ Health care staff serves as a direct source of medical information to the public.^63^ Therefore, it might be more difficult to gain this knowledge for women who are not exposed to health care staff.^64^ Furthermore, the higher rate of unemployment among women from the Gaza Strip versus the WBJ (63.7% *v* 25.8%) could also contribute to the knowledge difference.^65^ This may give more opportunities for women from the Gaza Strip to search and read the internet and other sources to enrich their knowledge about different health-related issues including BC.

The low level of awareness of BC risk factors in the Palestinian society provides new opportunities to introduce collaborations among different national and international BC awareness campaigns. Such health education programs may improve the utilization of screening services across Palestine. This could be facilitated by incorporating BC symptom awareness in these programs. Women eligible for BC screening were found to be more likely to recognize BC symptoms.^19^ This may drive these women to undergo BC screening, which will eventually lead to an increase in the rates of BC early detection. Our group has also found a significant association between higher recognition of BC symptoms and an earlier seeking for medical advice for a possible BC symptom (unpublished data). All this highlights the importance of establishing these integrated educational programs. Targeting communities with less educational opportunities and remote areas with little access to health care services should also be considered. Furthermore, extending the use of opportunistic health education delivered by health care professionals could improve awareness in the general population. Exploration of possible knowledge gaps and beliefs toward BC risk factors and screening among health care staff could be the basis for facility-based staff training and, thus, effective delivery of health education.

The main strength of this study is the community-based approach covering a wide geographical area of Palestine and reaching various communities. In addition, the large sample size and the high response rate facilitated a broad representation of the Palestinian population. This might have mitigated the limitation of using convenience sampling but does not guarantee the generalizability of the study findings to all women in Palestine. Another limitation could be the exclusion of patients or visitors to the oncology departments and those with medical backgrounds, which might have possibly reduced the number of participants with a presumably good awareness. However, their exclusion was meant to make this study more relevant as an assessment of the public awareness of BC.

In conclusion, the overall awareness of BC risk factors in the current study was suboptimal, with only 38.4% of the participants having good knowledge. In addition, only 3.3% of participants were aware of the age-related risk of getting BC, and about one quarter was aware of the lifetime risk of BC. These findings reinforce the continuing need for more BC risk factor awareness through conducting health education programs. Furthermore, effective programs for improving the health literacy of Palestinian women could include the equipment of health care professionals with knowledge and tools to inform women as contact with health care professionals appeared to have a positive impact on women's BC awareness.

## Figures and Tables

**TABLE A1:**
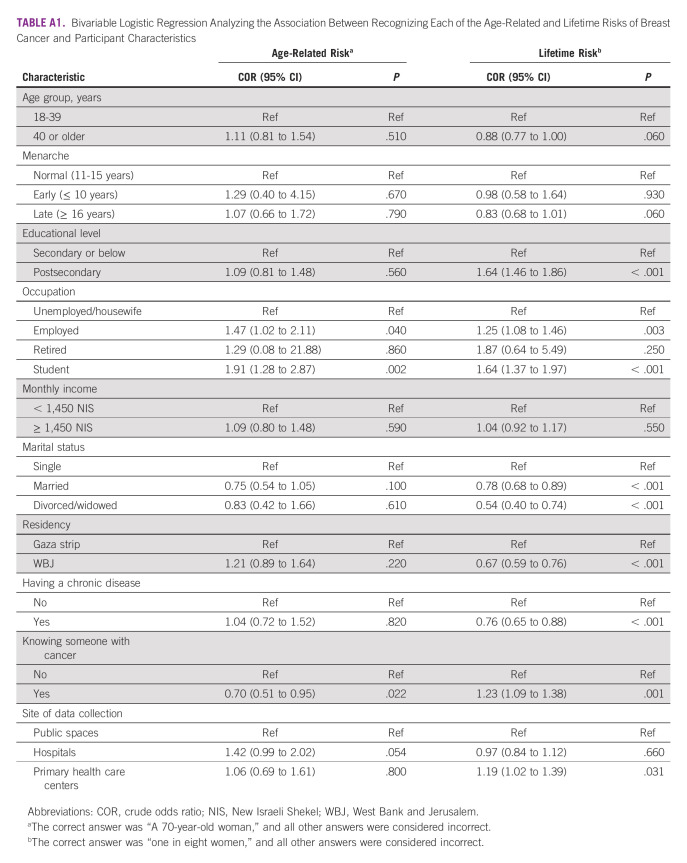
Bivariable Logistic Regression Analyzing the Association Between Recognizing Each of the Age-Related and Lifetime Risks of Breast Cancer and Participant Characteristics

**TABLE A2:**
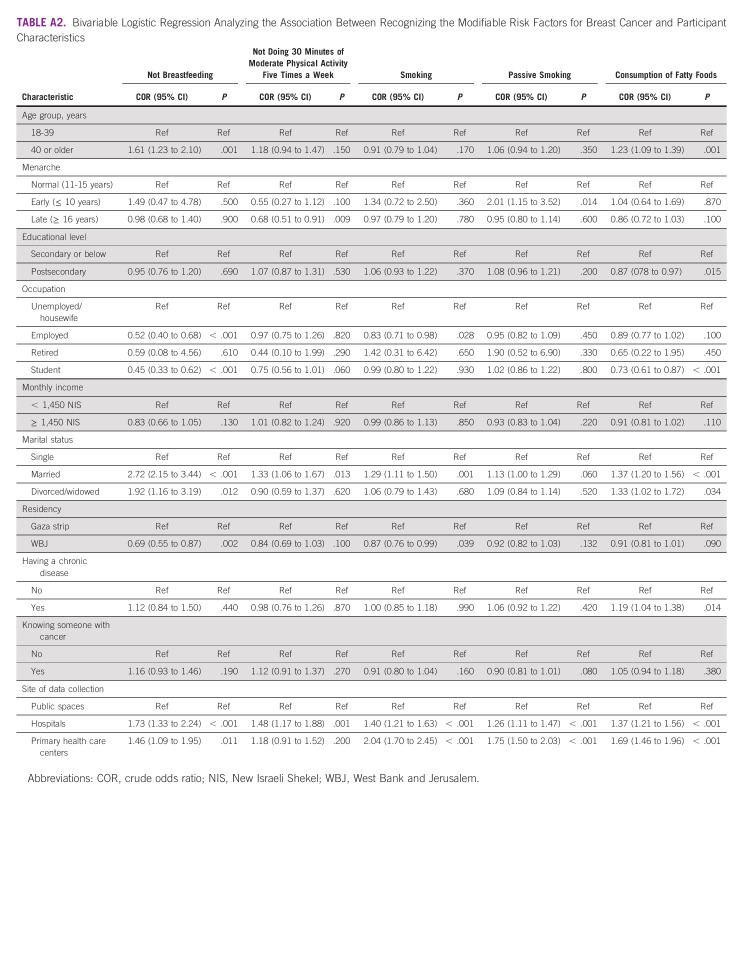
Bivariable Logistic Regression Analyzing the Association Between Recognizing the Modifiable Risk Factors for Breast Cancer and Participant Characteristics

**TABLE A3:**
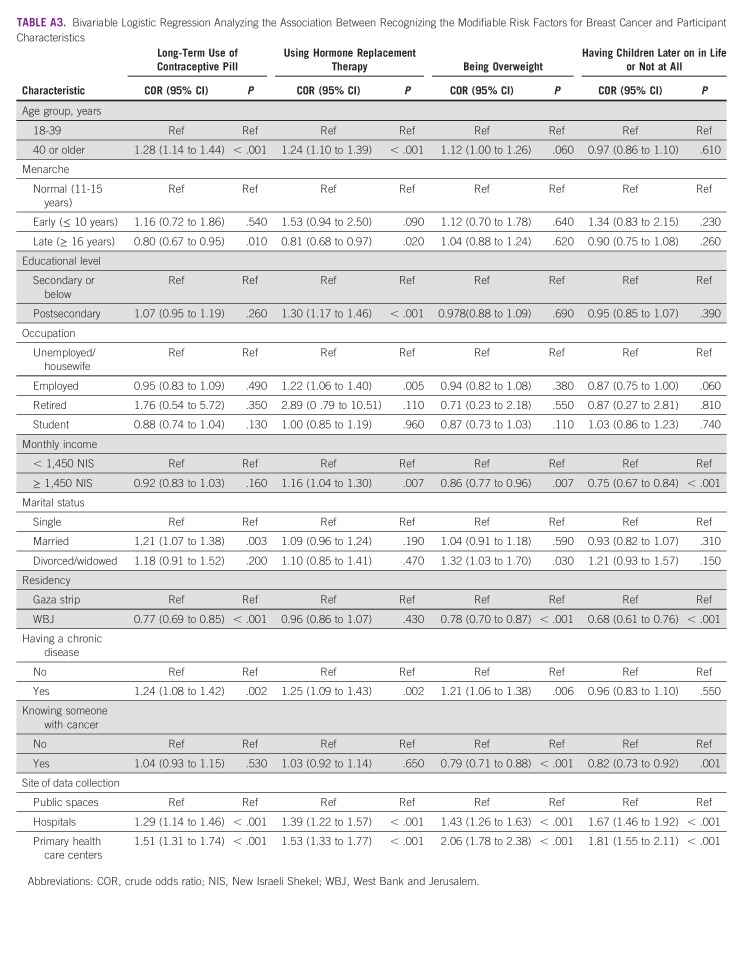
Bivariable Logistic Regression Analyzing the Association Between Recognizing the Modifiable Risk Factors for Breast Cancer and Participant Characteristics

**TABLE A4:**
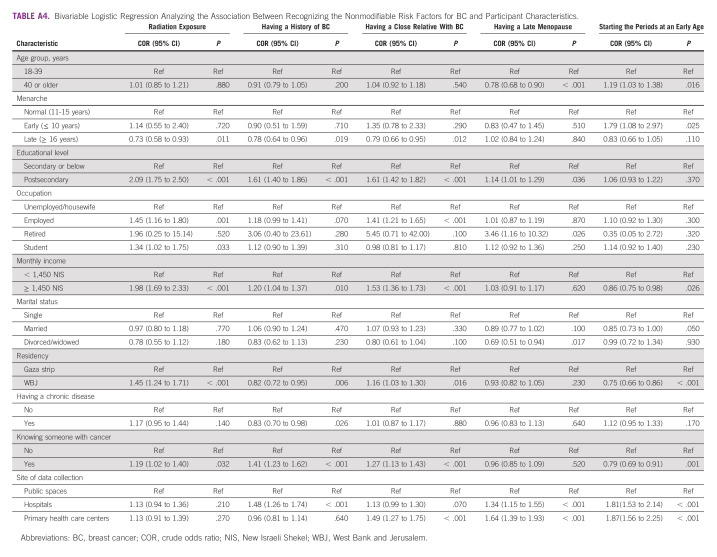
Bivariable Logistic Regression Analyzing the Association Between Recognizing the Nonmodifiable Risk Factors for BC and Participant Characteristics.
